# Supplementary material for: Specificity of AMPylation of the human chaperone BiP is mediated by TPR motifs of FICD
Source: Nat Commun. 2021 Apr 23;12:2426. doi: 10.1038/s41467-021-22596-0 (PMC8065156; doi:10.1038/s41467-021-22596-0)
Supplement: Supplementary file 1 — Supplementary Information [file 41467_2021_22596_MOESM1_ESM.pdf]

Supplementary Information

Specificity of AMPylation of the human chaperone BiP is mediated by TPR motifs of FICD

Authors

Joel Fauser[1,2], Burak Gulen[1,2], Vivian Pogenberg[1], Christian Pett[3], Danial Pourjafar-Dehkordi[4], Christoph Krisp[5], Dorothea Höpfner[1,2], Gesa König[1], Hartmut Schlüter[5], Matthias J. Feige[2,6], Martin Zacharias[4], Christian Hedberg[3,*], Aymelt Itzen[1,2,7,*]

Affiliations

[1] Department of Biochemistry and Signal Transduction, University Medical Center Hamburg-Eppendorf (UKE), 20246 Hamburg, Germany.

[2] Center for Integrated Protein Science Munich (CIPSM), Department Chemistry, Technical University of Munich, 85747 Garching, Germany.

[3] Chemical Biology Center (KBC), Institute of Chemistry, Umeå University, 90187 Umeå, Sweden.

[4] Physics Department T38, Technical University of Munich, 85748 Garching, Germany.

[5] Clinical Chemistry and Laboratory Medicine, Mass Spectrometric Proteomics, University Medical Center Hamburg-Eppendorf (UKE), 20246 Hamburg, Germany.

[6] Institute for Advanced Study, Technical University of Munich, 85748 Garching, Germany.

[7] Center for Structural Systems Biology (CSSB), University Medical Center Hamburg-Eppendorf (UKE), 22607 Hamburg, Germany.

*Corresponding authors: a.itzen@uke.de, christian.hedberg@umu.se

Supplementary Fig. 1

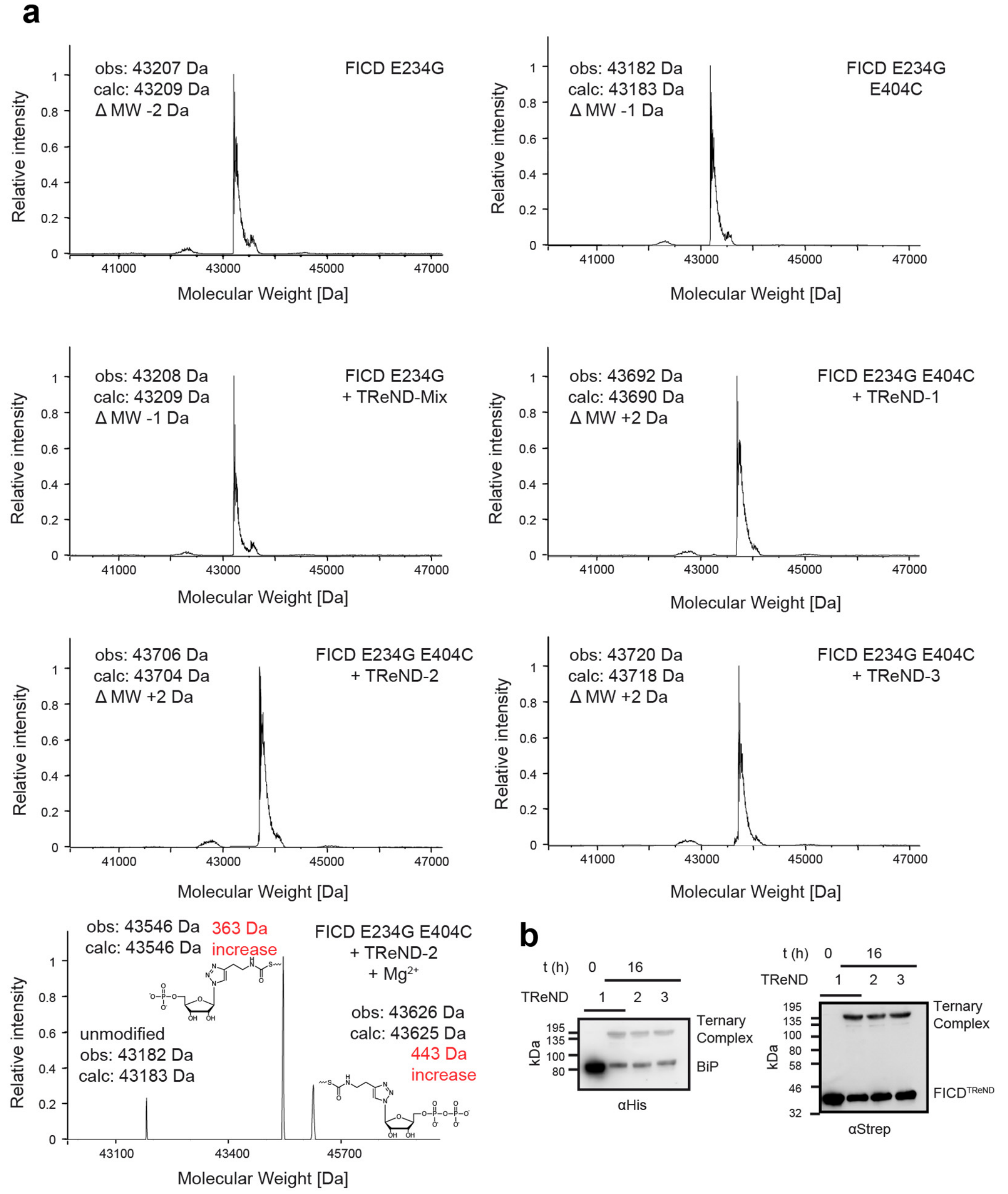

Supplementary Fig. 1. ***Intact mass spectrometry demonstrating regioselective reaction of TReNDs with FICD E234G E404C and verification of ternary complex formation.*** **a)** LC/HR-ESI-TOF-MS spectra of FICD 102-458 E234G and FICD 102-458 E234G E404C after incubation with different TReNDs. The mass deviation is indicated as Δ MW. For clarity reasons the ion spectrum including Mg was deconvoluted by the pMod algorithm while spectra of complexes without Mg^{2+} were deconvoluted by the maximum entropy algorithm. Herein, the mass deviation is –1 Da for unreacted FICD, 0 Da for $FICD^{TReND-2}$ in monophosphate state, and +1 Da for $FICD^{TReND-2}$ in diphosphate state. **b)** Ternary complex formation of Strep-FICD E234G E404C 102-458 and His-BiP 19-654. Western blot of covalently linked ternary complex with αHis and αStrep probes. Source data are provided as a Source Data file.

Supplementary Fig. 2

a

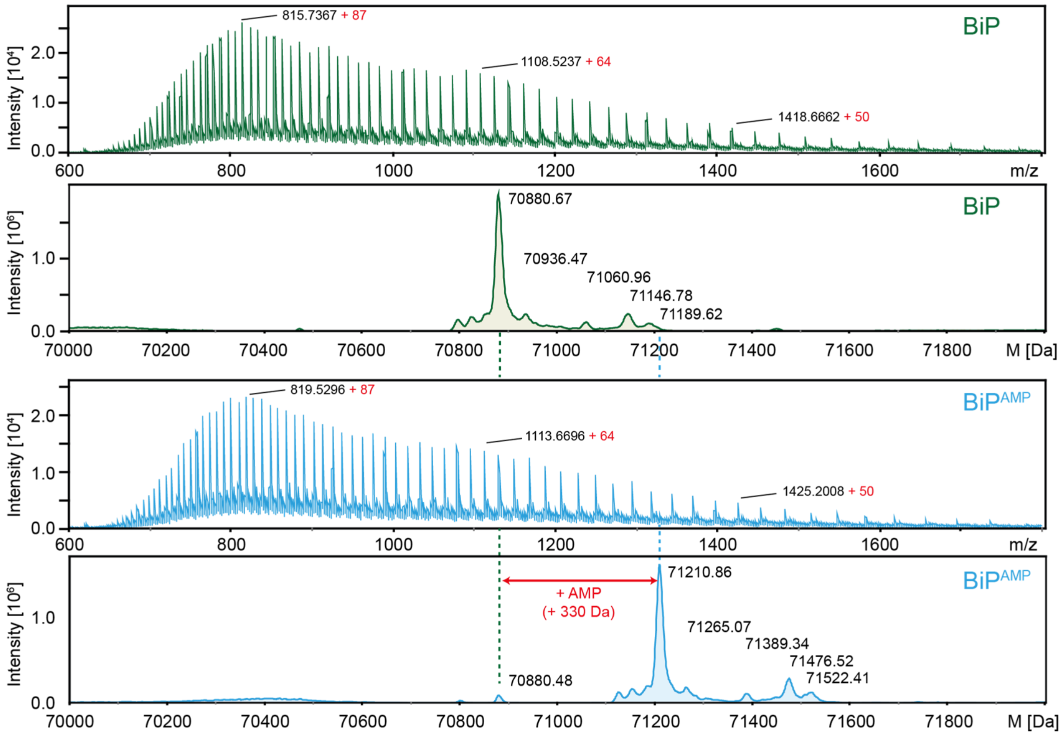

b

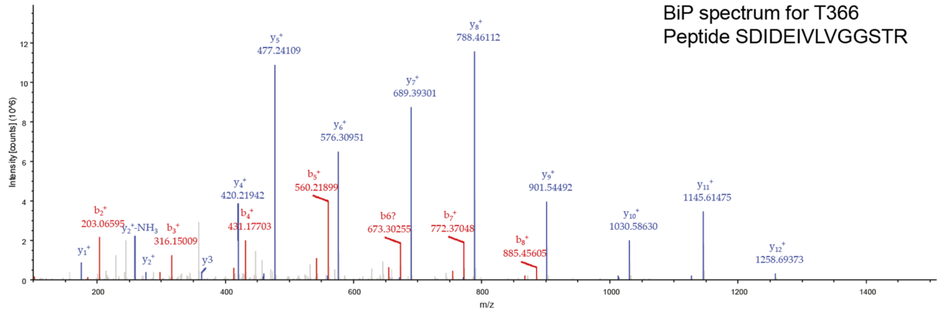

MS/MS spectrum of the T8-Phosphoadenosine modified peptide SDIDEIVLVGGSTR from BiP; charge: +2, monoisotopic m/z: 730.8829 (-0.44 ppm) Sequest HT XCorr: 4.08, Percolator q-Value: 7.3e-4, Percolator PEP:1.1e-4

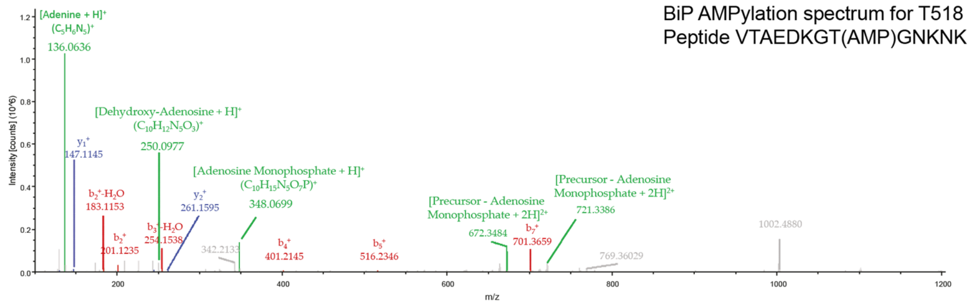

MS/MS spectrum of the T8-Phosphoadenosine modified peptide VTAEDKGTGNKNK from BiP; charge: +3, monoisotopic m/z: 564.2561 (-0.41 ppm) Sequest HT XCorr: 0.39, Percolator q-Value: 3.5e-3, Percolator PEP:2.8e-1

Supplementary Fig. 2. ***BiP is monoAMPylated at T518.*** a) Intact mass spectrometry of unmodified (green) and AMPylated BiP (blue). b) Peptide spectra of trypsin-digested BiP^{AMP} reveal that FICD modifies BiP specifically at T518. Peptides comprising T366 were exclusively found unmodified.

Supplementary Fig. 3

a

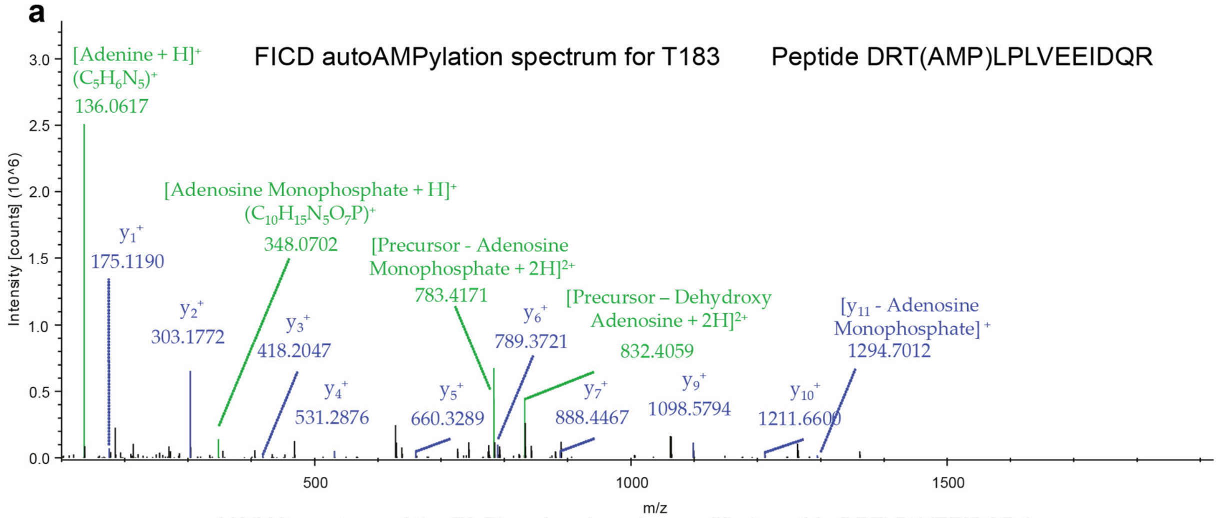

MS/MS spectrum of the T3-Phosphoadenosine modified peptide DRTLPLVEEIDQR from FICD (Protein adenylyltransferase FICD); charge: +3, monoisotopic m/z: 638.3016 (-0.58 ppm), Sequest HT XCorr: 1.75, Percolator q-Value: 0, Percolator PEP: 0.0008

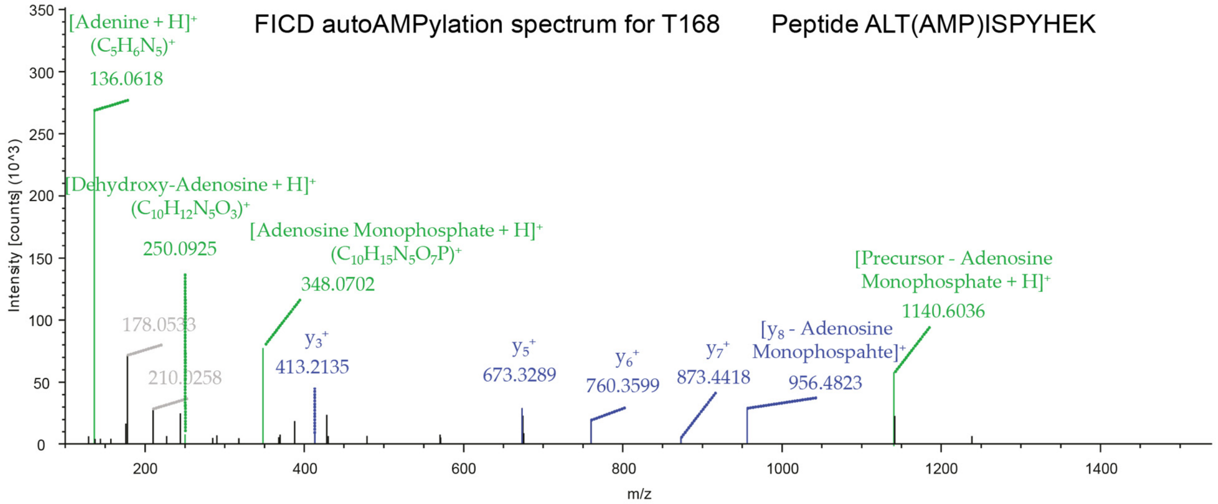

MS/MS spectrum of the T3-Phosphoadenosine modified peptide ALTISPYHEK from FICD (Protein adenylyltransferase FICD); charge: +2, monoisotopic m/z: 744.3386 (1.33 ppm), Sequest HT XCorr: 0.51, Percolator q-Value: 0.0024, Percolator PEP: 0.09.

b

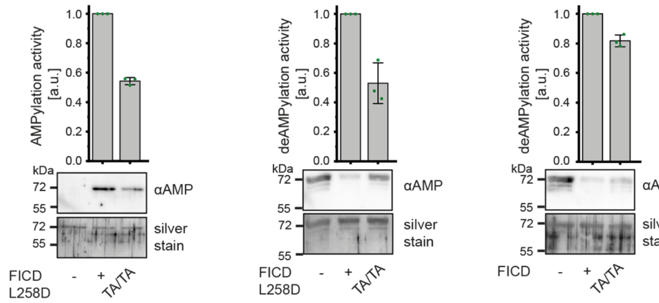

Supplementary Fig. 3. ***FICD 102-458 E234G is autoAMPylated at T168 and T183.*** **a)** Peptide LC-MS/MS spectra of autoAMPylated FICD 102-458 E234G. Peptide spectra of trypsin-digested FICD reveal two AMPylation sites T168 and T183. **b)** Effect of $T168_{FICD}$ and $T183_{FICD}$ on BiP AMPylation and deAMPylation. AMPylation of BiP 19-654 WT by FICD L258D (+) and FICD T168A T183A L258D (TA/TA) (left panel). DeAMPylation of BiP 19-654 WT by FICD L258D (+) and FICD T168A T183A L258D (TA/TA) (middle panel). DeAMPylation of BiP 19-654 WT by FICD (+) and FICD T168A T183A (TA/TA) (right panel). Please note, that the substitution of the automodified threonines by alanines leads to decreased AMPylation and deAMPylation activity. The reason for these effects is unclear and may result from structural perturbation of FICD T168A T183A L258D / FICD T168A T183A or the absence of a possibly stimulating effect of automodification. The experiment was performed in three independent replicates. Data are presented as mean values +/- standard deviation. The abbreviation 'a.u.' represents 'arbitrary units'. Source data are provided as a Source Data file.

Supplementary Fig. 4

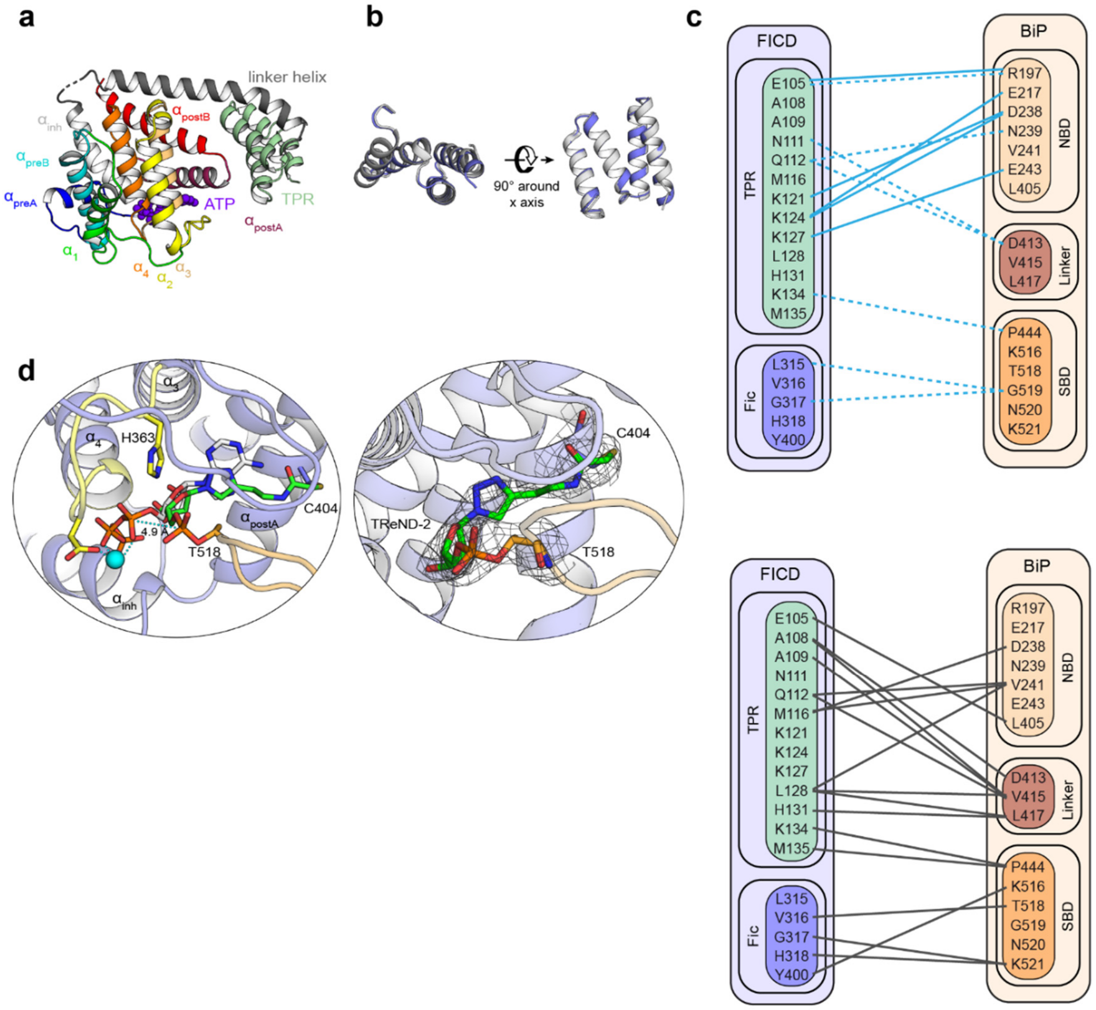

Supplementary Fig. 4. ***Structural analysis of the covalently linked FICD:BiP complex.*** **a)** Detailed representation of secondary structure elements of FICD 102-458 (PDB 4U07). The N-terminal TPR motifs (green) connect to the Fic domain via the linker helix (grey). The helices of the Fic domain are colored in rainbow (from α_{inh} (white) and α_{preA} (blue) to α_{postB} (red)). ATP is displayed in purple. **b)** Structural integrity of the TPR motifs in the complex structure as shown by superimposition of the TPR motifs (residues 104-171) of FICD from the complex structure (purple) and isolated FICD (grey; PDB: 6I7G). **c)** Overall interaction profile of FICD with BiP as determined from the complex structure. **d)** Left panel: Detailed view on the positioning of TReND-2 (green) within the ATP-binding pocket of FICD (purple) in complex with BiP (orange). The Fic motif is highlighted in yellow. The structure of isolated FICD L258D:ATP (PDB 6I7K) was overlaid to the structure of complexed FICD. For clarity only the ATP (white) and the Mg^{2+} (cyan) from isolated FICD:ATP structure is shown. The distance of the α-phosphates of regular ATP (from isolated FICD:ATP) and TReND-2 (from the complex) was measured to 4.9 Å (dashed line in cyan). Note, that in the AMPylation competent position of ATP (as determined by PDB 6I7K), the α-phosphate is positioned close to D367 (yellow sticks) to coordinate Mg^{2+} (as illustrated by the dashed line in cyan). Right panel: Unbiased electron density map (OMIT) of the covalent linker TReND-2. The displayed map was constructed with a sigma 3.0 and carve distance of 1.6 Å.

Supplementary Fig. 5

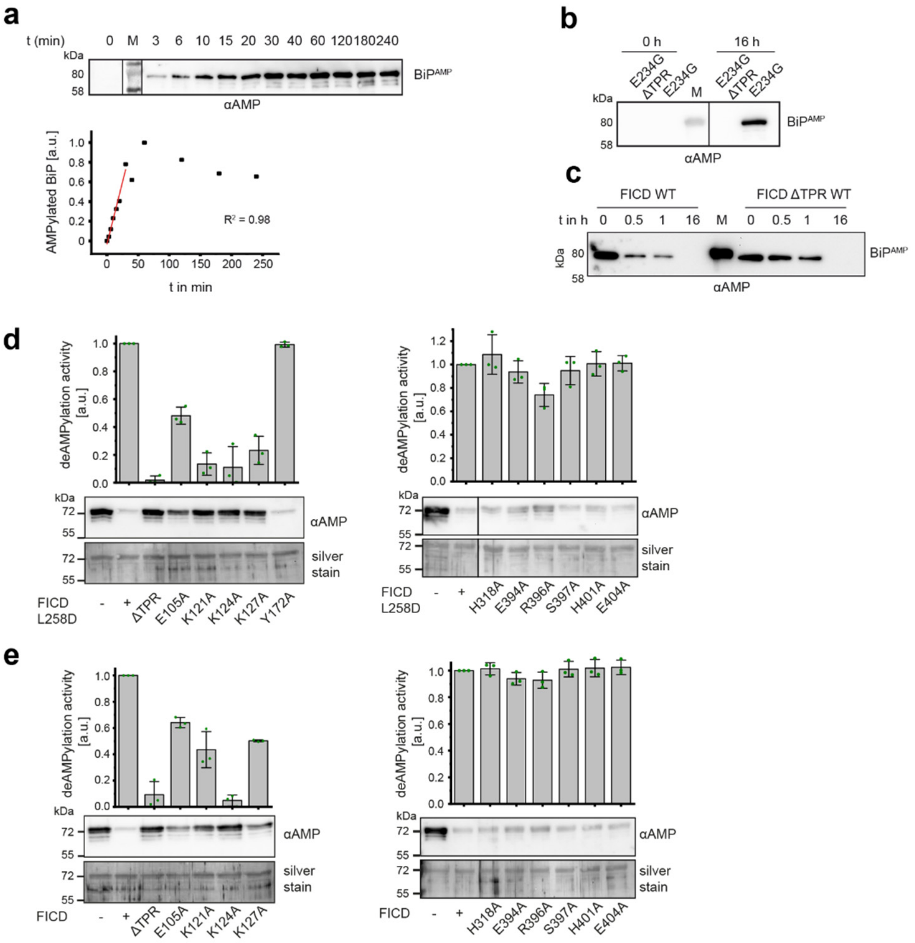

Supplementary Fig. 5. ***AMPylation and deAMPylation by FICD L258D and FICD.*** **a)** AMPylation of BiP 19-654 by FICD 102-458 L258D visualized by western-blotting using an αAMP-specific antibody. The linear increase of AMPylated BiP over time (until t = 30 min) indicates negligible deAMPylation activity under the chosen experimental conditions. **b)** AMPylation of BiP 19-654 by FICD 102-458 E234G ('E234G') and FICD 187-458 E234G ('FICD ΔTPR') visualized by western-blotting using an αAMP-specific antibody. The experiment was performed three times independently with similar results. **c)** DeAMPylation of BiP by FICD 102-458 WT ('FICD WT') and FICD 187-458 WT ('FICD ΔTPR WT') visualized by western-blotting using an αAMP-specific antibody. The experiment was performed two times independently with similar results. **d)** DeAMPylation of BiP^{AMP} by FICD L258D and corresponding alanine substitutions visualized by western-blotting using an αAMP-specific antibody. The reaction was performed in presence of 0.5 mM ApNHpp (non-transferable ATP analogue) and stopped after 5 min by addition of Laemmli buffer. The relative deAMPylation activity describes the normalized difference of BiP^{AMP} with and without (-) enzyme. Since some mutants exhibited very low deAMPylation activity (ΔTPR, K124A), in some replicates negative values were obtained after subtraction and set to '0'. The experiment was performed in three independent replicates. Data are presented as mean values +/- standard deviation. **e)** DeAMPylation of BiP^{AMP} by FICD and corresponding alanine substitutions visualized by western-blotting using an αAMP-specific

antibody. The experiment was performed in three independent replicates. Data are presented as mean values +/- standard deviation. The same experimental procedures as in ‘d’ were applied. The abbreviation ‘a.u.’ represents ‘arbitrary units’. Source data are provided as a Source Data file.

Supplementary Fig. 6

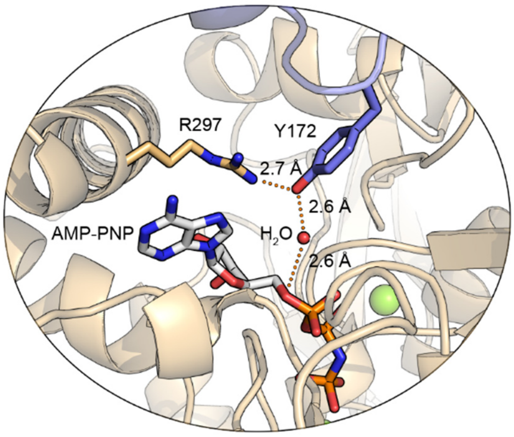

Supplementary Fig. 6 ***Crystal contact of Y172$_{FICD}$ with the nucleotide in the nucleotide binding pocket of BiP.*** Detailed view on crystal contacts of R297$_{BiP}$, Y172$_{FICD}$ and AMP-PNP, bound to BiP. FICD is colored purple and BiP is colored wheat.

Supplementary Fig. 7

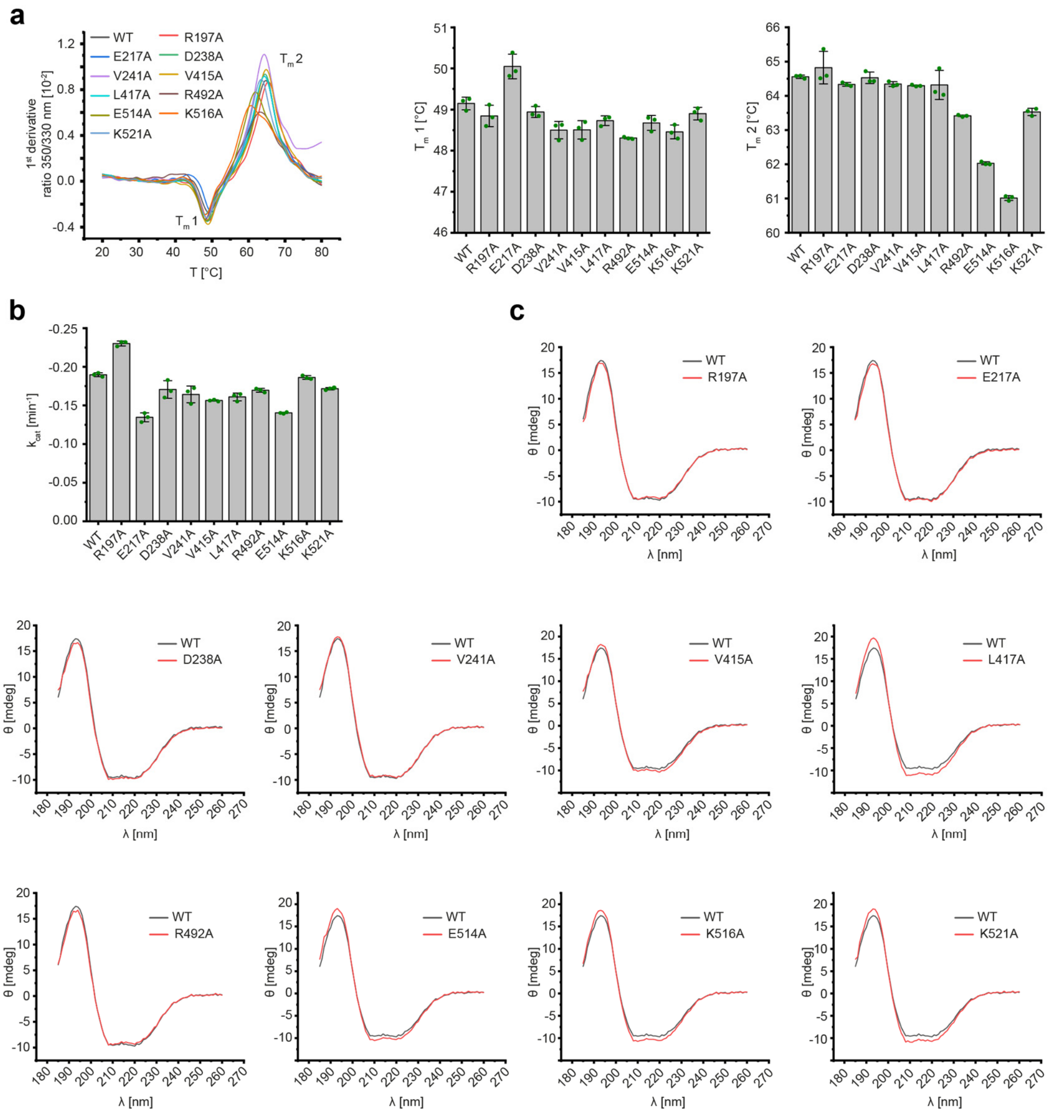

Supplementary Fig. 7. ***Biochemical and biophysical characterization of the BiP mutants.*** **a)** The melting points of the BiP constructs was determined via NanoDSF. Two distinct melting points (T_m1 for NBD and T_m2 for SBD) were observed as displayed in the first derivative of the fluorescence ratio 350/330 nm (left panel). The melting points of all BiP constructs are summarized in the middle (T_m1) and right panel (T_m2). The experiment was performed in three independent replicates. Data are presented as mean values +/- standard deviation. **b)** Determination of steady state ATPase kinetics were obtained using an ATP regenerating system. ATP hydrolysis results in the consumption of NADH yielding a negative k_{cat} value. The experiment was performed in three independent replicates. Data are presented as mean values +/- standard deviation. **c)** For all BiP constructs a CD spectrum ranging from 185 to 260 nm was obtained. The ellipticity θ is displayed in the unit of mdeg. The experiment was performed in three independent replicates with similar results. Source data are provided as a Source Data file.

Supplementary Fig. 8

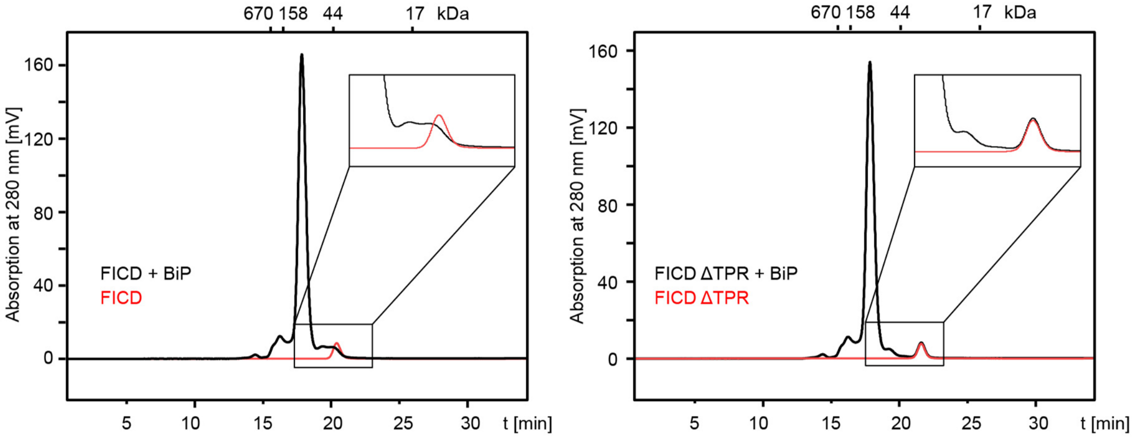

Supplementary Fig. 8. ***The TPR motifs are important for BiP binding.*** 5 µM FICD L258D with (102-458, left panel) and without (187-458, right panel) the TPR motifs were added to 100 µM BiP 19-654 T229A T518A in presence of 5 mM ATP. The spectra suggest that FICD binding to BiP is dependent on the TPR motifs. Source data are provided as a Source Data file.

Supplementary Fig. 9

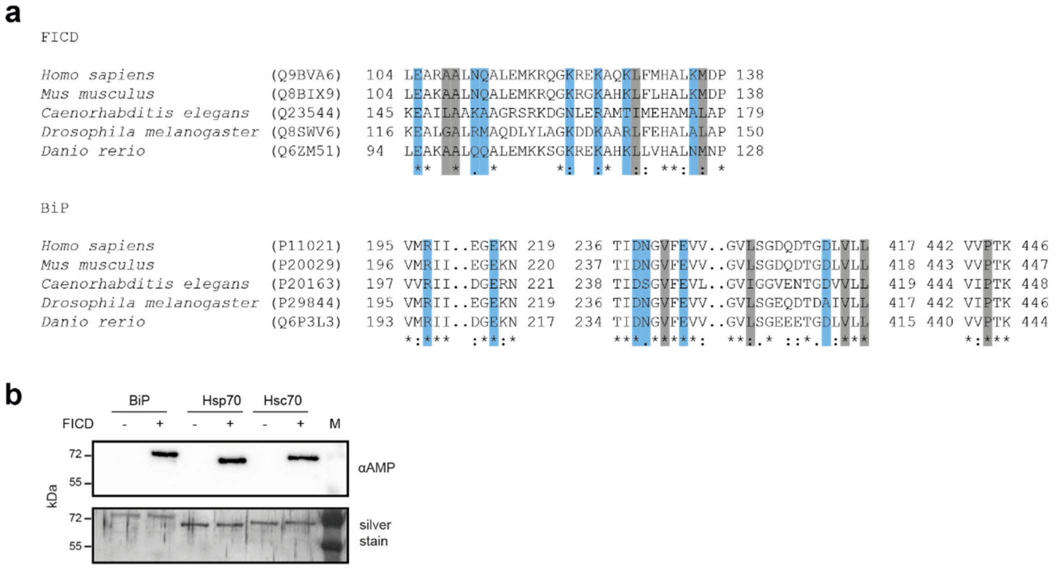

Supplementary Fig. 9. ***The binding mode of the TPR motifs of FICD is conserved.*** a) Sequence alignment of FICD and BiP from different species using the Clustal Omega Multiple Sequence Alignment tool. The interacting residues within FICD and BiP are highlighted in blue (hydrophilic interactions) and grey (hydrophobic interactions). b) FICD AMPylates human BiP, Hsp70 and Hsc70 as detected by western blot with an AMP-specific antibody. The reaction was stopped after 2 h by addition of Laemmli buffer. The experiment was performed three times independently with similar results. Source data are provided as a Source Data file.

Supplementary Fig. 10

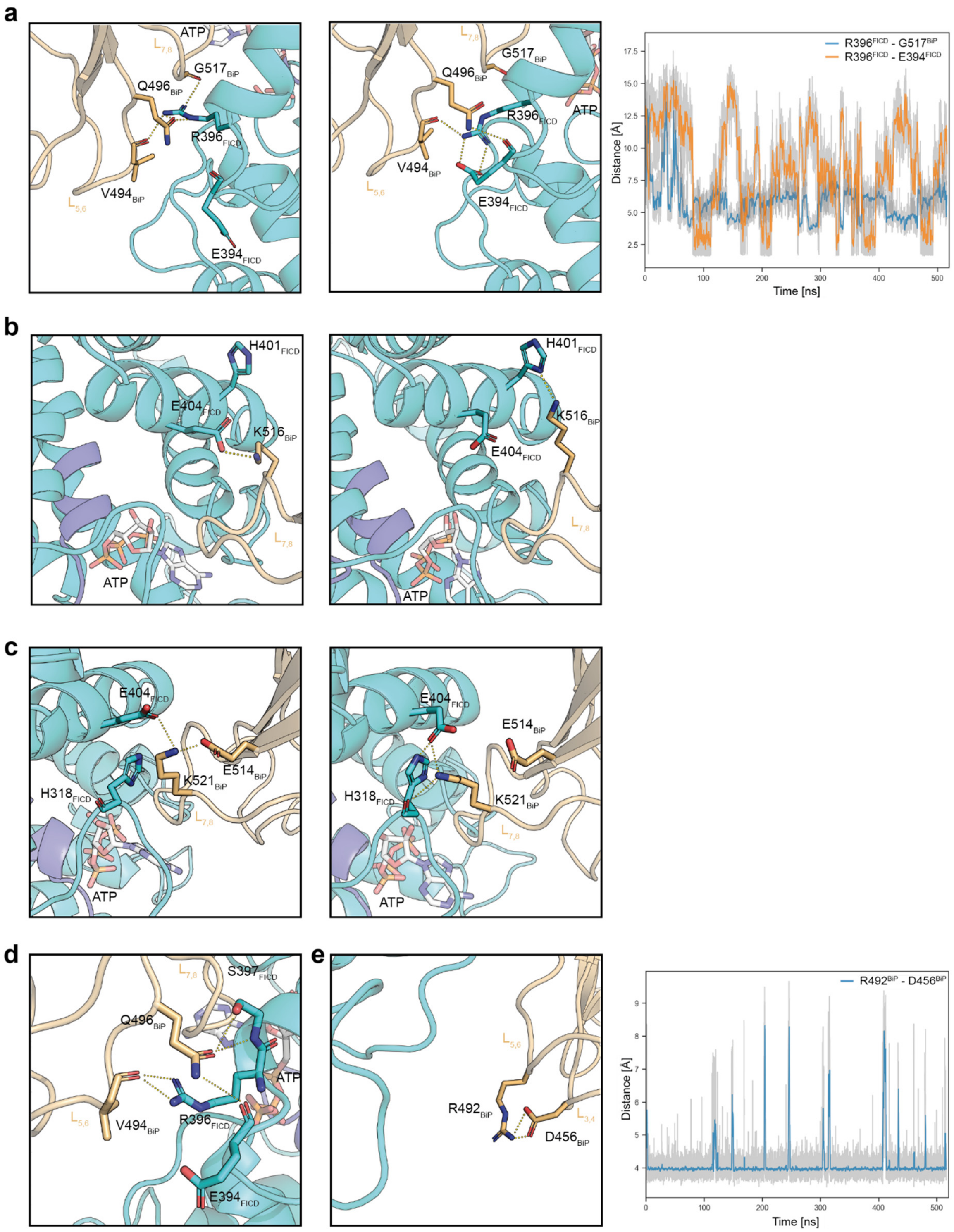

Supplementary Fig. 10. ***The interactions of the SBD and Fic domain as determined by molecular dynamics simulations.*** **a)** The left snapshot illustrates the interaction of R496$_{FICD}$ with G517$_{BiP}$, V494$_{BiP}$ and Q496$_{BiP}$**.** This intimate network of R396$_{FICD}$ may explain the fundamental defect of FICD R492A in BiP AMPylation. In addition, substitution of one of R396$_{FICD}$'s interaction partner by alanine (Q496A$_{BiP}$) leads to reduced (-52%) AMPylation activity. Interestingly, substitution of E394$_{FICD}$ by alanine leads to slightly elevated AMPylation activity (**Fig. 3**), which could be explained by its intramolecular binding to R396$_{FICD}$, which directly competes with binding to the BiP residues (right snapshot). In the right panel, atomic distances versus simulation time illustrate the presence of intermolecular bond between R396$_{FICD}$ - G517$_{BiP}$, indicated by the atomic distances between NH1 and O atoms, and the intramolecular bond between R396$_{FICD}$- E394$_{FICD}$, indicated by the distance between NH2 and OE2 atoms. The grey lines show the distances obtained in 20ps intervals. The orange and blue lines illustrate the moving average calculated over a 1ns window. **b)** The two snapshots indicate two putative interaction partners of K516$_{BiP}$, which proved crucial for AMPylation (-99%). However, mutation of its putative interaction partners H401 and E404 (left and right snapshots) reduced AMPylation by just 60% and 75%, respectively. **c)** The left snapshot illustrates the interaction of K521$_{BiP}$ with H318$_{FICD}$ and E404$_{FICD}$. While the biochemical data proved the contribution of H318$_{FICD}$ and E404$_{FICD}$ to BiP AMPylation, mutation of K521 to alanine slightly elevated BiP AMPylation, possibly due to relieved electrostatic repulsion with H318$_{FICD}$. **d)** Illustration of the interaction of S397$_{FICD}$ with Q496$_{BiP}$. The rather small decrease in AMPylation upon S397$_{FICD}$ substitution can be explained by the finding of Q496$_{BiP}$ interacting with both the side chain and the backbone amine of S397$_{FICD}$.**e)** Snapshot illustrating the positioning of R492$_{BiP}$ and its interaction with D456$_{BiP}$. Based on the distance of NH1 (R492$_{BiP}$) to the O atom (D456$_{BiP}$) the right panel illustrates, that the interaction is maintained most of time during the simulation and R492$_{BiP}$ therefore not available for interactions with FICD.

Supplementary Fig. 11

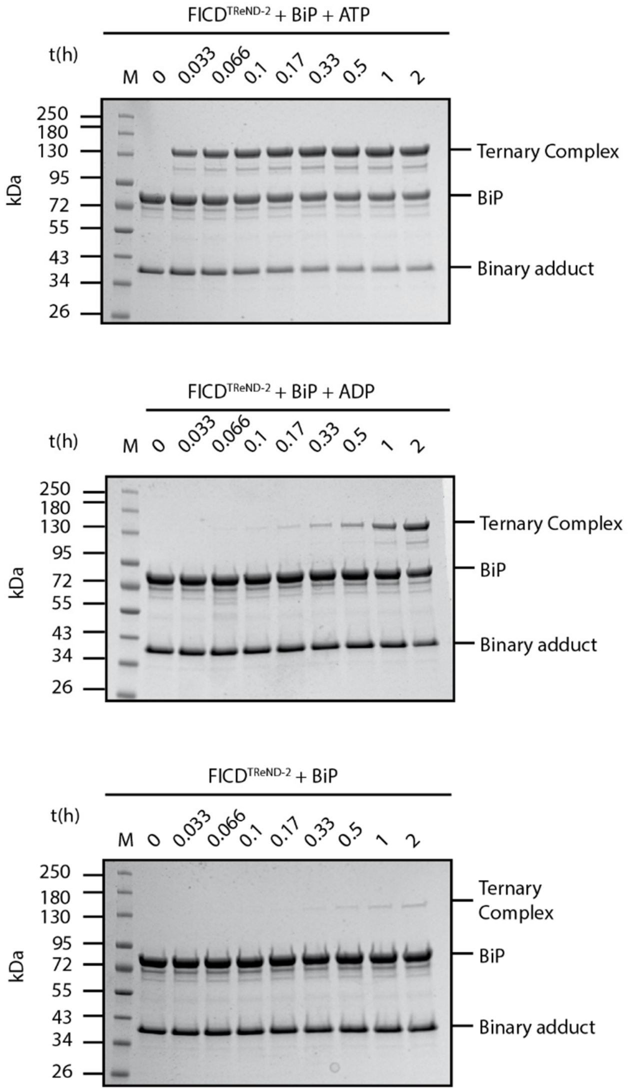

Supplementary Fig. 11. The TPR motifs mediate specificity towards ATP-bound BiP. Full SDS-PAGE gels displaying ternary complex formation in absence or presence of ADP and ATP. Source data are provided as a Source Data file.

Supplementary Fig. 12

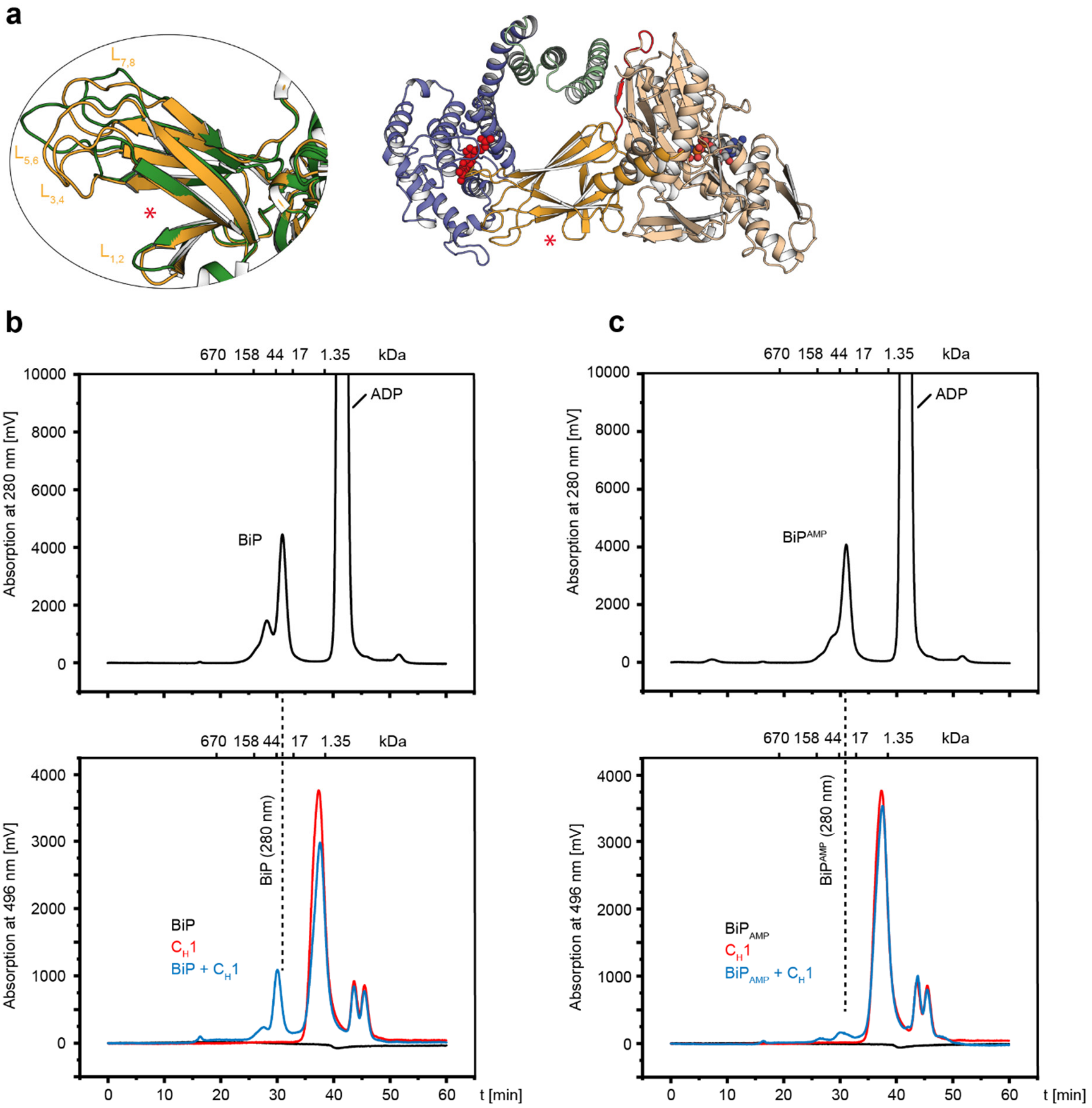

Supplementary Fig. 12. ***Binding of BiP to the unfolded protein substrate C_H1 is impaired upon AMPylation.*** a) Left panel: Detailed view on the SBD of complexed BiP (orange) overlaid to BiP:ATP (green, PDB: 5E84). The cleft where protein substrates bind is open and indicated by the red star. Right panel: Position of the substrate binding cleft in the complex structure. The cleft where protein substrates bind is indicated by the red star. b) Binding of unmodified BiP (10 µM) to the unfolded protein substrate C_H1 (2.5 µM). The upper panel displays BiP trace at 280 nm whereas the lower panel indicates BiP binding to FITC labeled-C_H1 at 496 nm. c) Binding of AMPylated BiP (BiP^{AMP}) (10 µM) to the unfolded protein substrate C_H1 (2.5 µM). The upper panel displays BiP^{AMP} trace at 280 nm whereas the lower panel indicates BiP^{AMP} binding to FITC-labeled C_H1 at 496 nm. Source data are provided as a Source Data file.

Supplementary Fig. 13

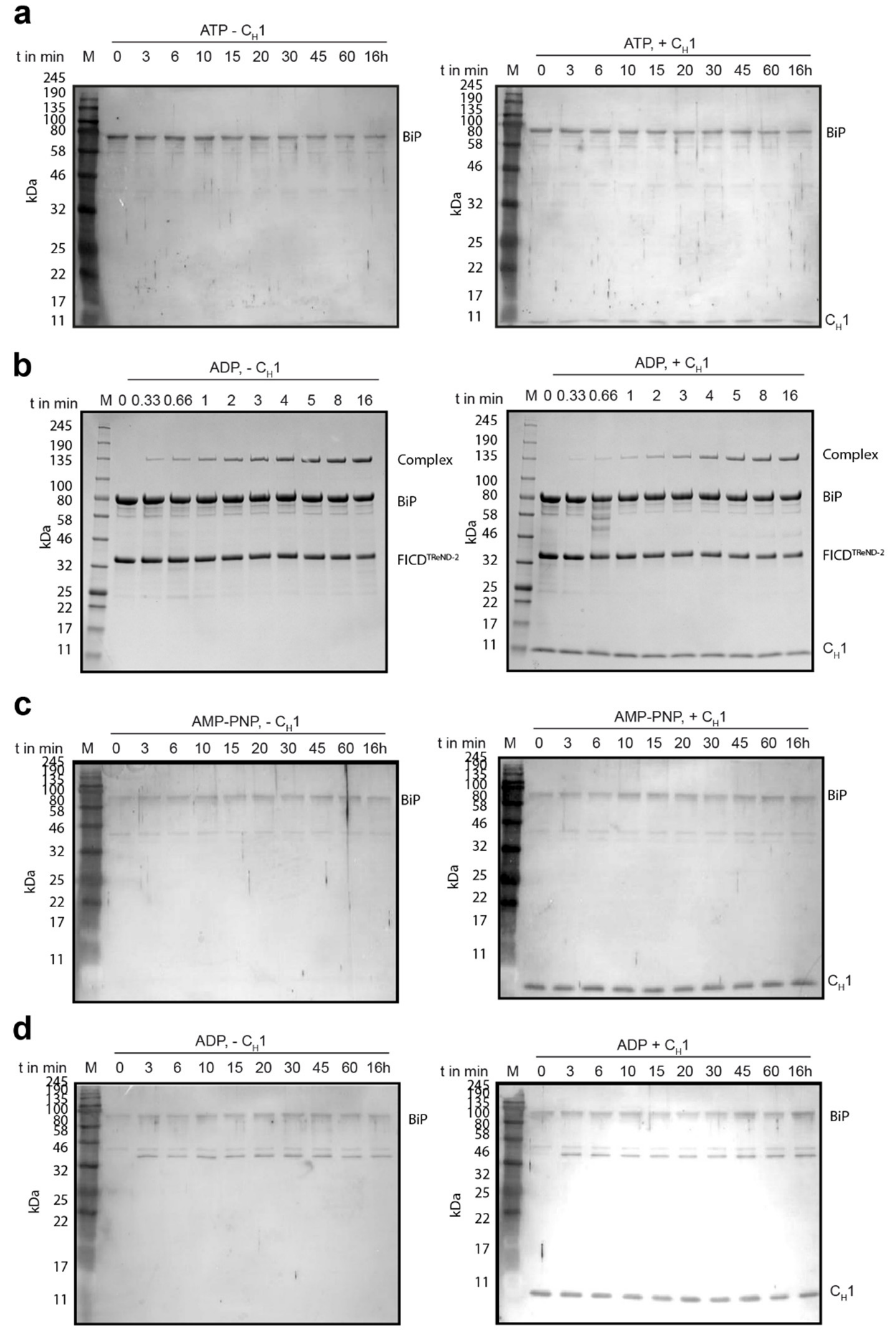

Supplementary Fig. 13. ***Influence of the unfolded protein substrate C_H1 on BiP AMPylation an deAMPylation.*** **a)** Silver stained blots of FICD mediated AMPylation of BiP in presence or absence of C_H1. **b)** Full SDS-PAGE gel of ternary complex formation with FICD and BiP:ADP in presence or absence of C_H1. **c)** Silver stained blots of FICD mediated deAMPylation of BiP:AMP-PNP in presence or absence of C_H1. **d)** Silver stained blots of FICD mediated AMPylation of BiP:ADP in presence or absence of C_H1. Source data are provided as a Source Data file.

Supplementary Fig. 14

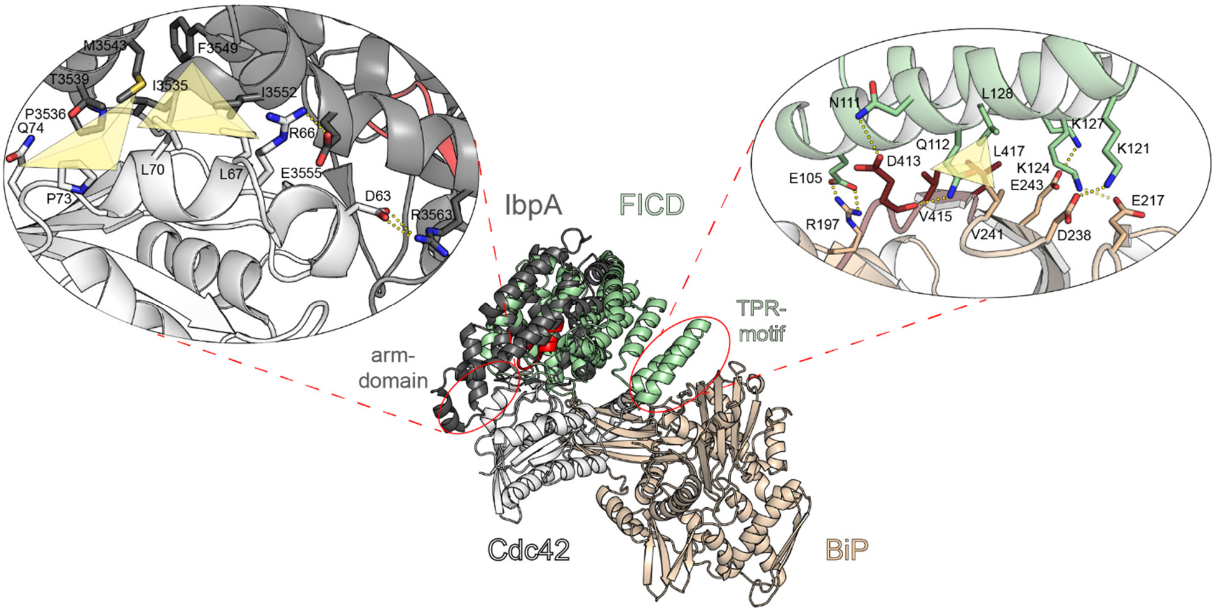

Supplementary Fig. 14. *Comparison of the IbpA:Cdc42 complex with the FICD:BiP complex.* The Fic-motifs (red) of IbpA in IbpA:Cdc42 (PDB: 4ITR) (grey:white) and FICD in FICD:BiP (green:wheat) were superimposed to highlight the relative orientation of the adjacent arm domain of IbpA and the TPR domain of FICD. For clarity, only the most prominent interacting residues are highlighted in the detailed comparisons of the TPR-based and arm-domain based interfaces (circles). The hydrophobic interfaces are displayed as yellow pyramids.

Supplementary Fig. 15

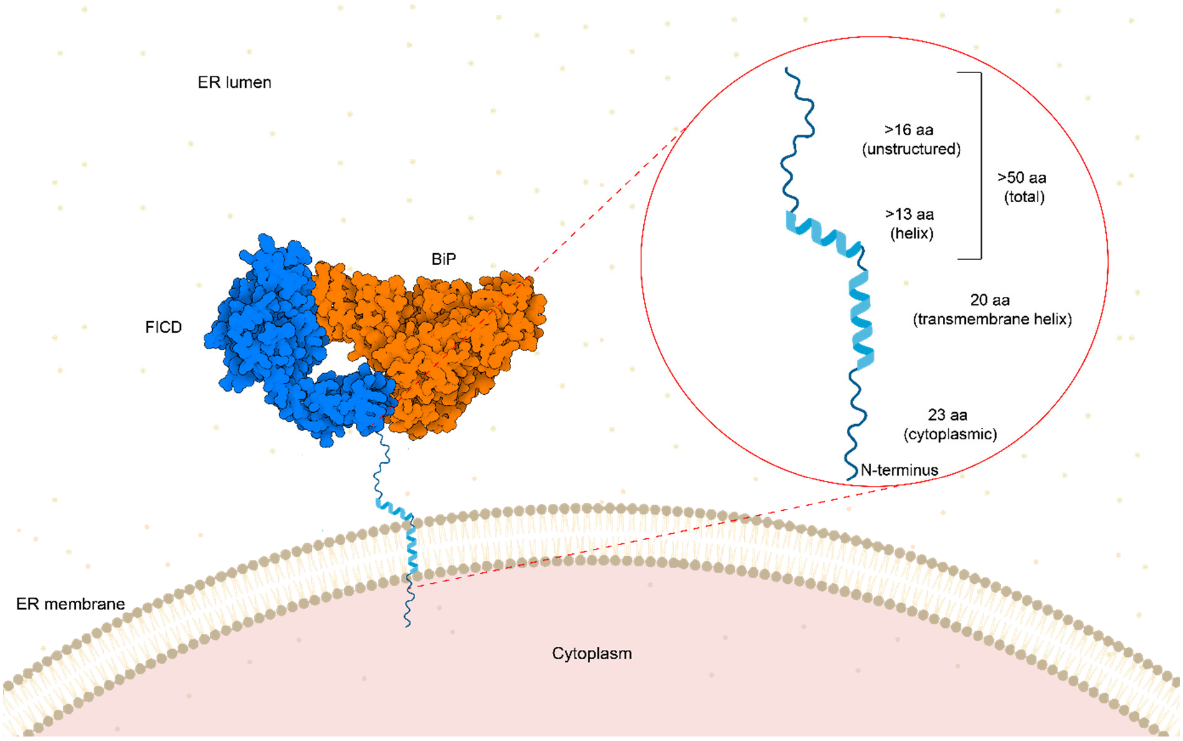

Supplementary Fig. 15. ***Schematic representation of the FICD:BiP complex associated to the ER membrane.*** BiP (orange) is bound to FICD (blue). The N-terminal part of FICD is predicted to bear a cytoplasmic tail, followed by a transmembrane domain that anchors the protein to the ER membrane (Uniprot). In between the transmembrane domain and the TPR motif that is involved in BiP recognition are more than 50 amino acids (aa), partially structured as helix, partially unstructured as predicted by JPred[1].

Supplementary Fig. 16

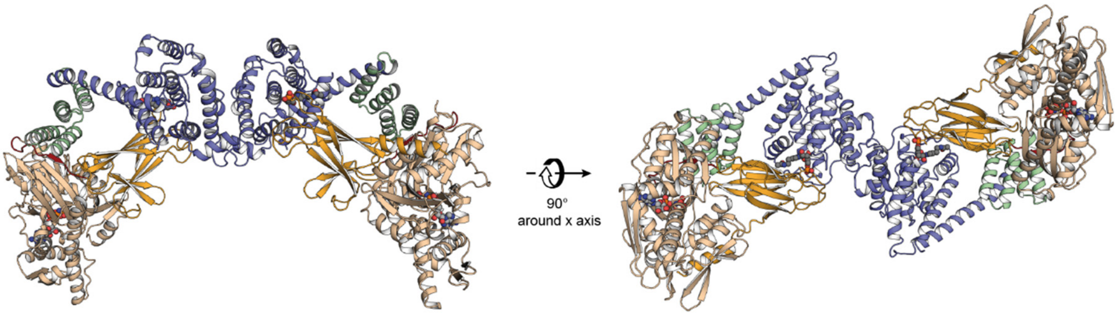

Supplementary Fig. 16. ***Model of a putative deAMPylation complex.*** FICD of the complex structure was overlaid on each chain of dimeric FICD (PDB: 4U04). Color coding as in Fig 2a. FICD is represented in purple (Fic domain) and green (TPR motifs). BiP is colored in wheat (nucleotide binding domain, NBD) and orange (substrate binding domain, SBD).

Supplementary Table 1

Data collection and refinement statistics.

Data collection	
**Wavelength (Å)**	0.976250
**Resolution range (Å)**	83.47 - 2.64 (2.77 - 2.64)
**Space group**	P 4_1
**Unit cell dimensions (Å)**	83.47 83.47 169.48<br>90 90 90
**Total reflections**	127291 (17378)
**Unique reflections**	34024 (4518)
**Multiplicity**	3.7 (3.8)
**Completeness (%)**	100 (100)
**Mean I/σ(I)**	10.1 (1.9)
**Wilson B-factor**	59.6
**R_{merge}**	0.057(0.567)
**$CC_{1/2}$**	0.99 (0.72)

Refinement and model statistics	
**R_{work}**	0.192
**R_{free}**	0.235
**Number of non-hydrogen atoms:**	6858
**macromolecules**	6718
**ligands**	66
**solvent**	74
**RMSD bonds (Å)**	0.002
**RMSD angles (˚)**	0.400
**Ramachandran:**	
**Ramachandran favored (%)**	98.5
**Ramachandran allowed (%)**	1.5
**Ramachandran outliers (%)**	0.0
**Average B-factor (Å^2)**	73.5
**macromolecules**	73.8
**ligands**	65.0
**solvent**	53.3

Statistics for the highest-resolution shell are shown in parentheses.

Supplementary Table 2

Supplementary Table 2. Primers for FICD constructs and mutagenesis

Primers for FICD constructs and mutagenesis		Sequence 5' - 3'
FICD E234G F	Fw	GTGGCCATCGgGGGCAACACC
FICD E234G R	Rv	TGTGTGGTAGATGTGATGGTAGTAGG
FICD L258D F	Fw	CGGGAAGAGCgacGAGGAGCAGAACGAGGTCATAGGC
FICD L258D R	Rv	GGCACGGCGTAGCGGGTC
FICD H363A F	Fw	CGTTTACATCgccCCTTTCATTGATG
FICD H363A R	Rv	AGTTTATAATGGGCTAAGG
FICD H319C F	Fw	ggtcctggtcggacacTGcatccctccccatccgcag
FICD H319C R	Rv	tggggagggatgCAgtgtccgaccaggacctgtgttg
FICD N407C F	Fw	gaagctgccTGcgagggcgacgtgaggcctttcattc
FICD N407C R	Rv	ggcctcacgtcgccctcgCAggcagcttccaacacgtg
FICD L403C F	Fw	CTACCACGTGtgcGAAGCTGCCAACG
FICD L403C R	Rv	TAGTCGGACCGCTGCTCC
FICD E404C F	Fw	CCACGTGTTGtgcGCTGCCAACGAGG
FICD E404C R	Rv	TAGTAGTCGGACCGCTGC
FICD T168A F	Fw	CAGAGCATTGgcaATCTCACCCTAC
FICD T168A R	Rv	GTGTACAAGTAGTCCGCC
FICD T183A F	Fw	CCGCGATCGGgcaCTGCCTCTTG
FICD T183A R	Rv	TTGACCAGTGCTTTCTCATGGTAGGG
FICD 102 -445 F	Fw	caggagtccaagctcagctaattaagcttagggttgggcttctggcagtg
FICD 102 -445 R	Rv	taagcttaattagctgagcttggac
FICD 187 -445 F	Fw	gtggaagagatcgaccag
FICD 187 -445 R	Rv	GCCCTGAAAATAAAGATTCTCATC
FICD E105A F	Fw	GGGCAAGTTGgccGCCAGAGCTG
FICD E105A R	Rv	TGAAAATAAAGATTCTCATCAGCC
FICD K121A F	Fw	GCGCCAGGGCgccCGGGAAAAAG
FICD K121A R	Rv	TTCATCTCCAGGGCCTGG
FICD K124A F	Fw	CAAGCGGGAAgccGCCCAAAAGCTCTTCATG
FICD K124A R	Rv	CCCTGGCGCTTCATCTCC
FICD K127A F	Fw	AAAAGCCCAAgccCTCTTCATGCACGCCCTCAAGATGGACC
FICD K127A R	Rv	TCCCGCTTGCCCTGGCGC
FICD Y172A F	Fw	CATCTCACCCgccCATGAGAAAGC
FICD Y172A R	Rv	GTCAATGCTCTGGTGTAC
FICD H401A F	Fw	CGACTACTACgccGTGTTGGAAGCTGCCAACG
FICD H401A R	Rv	GACCGCTGCTCCTTGCGG
FICD H318A F	Fw	CCTGGTCGGAgccCACATCCCTC
FICD H318A R	Rv	ACCTGTGTTGTCCGAAAC
FICD E404A F	Fw	CCACGTGTTGgctGCTGCCAACG
FICD E404A R	Rv	TAGTAGTCGGACCGCTGC
FICD S397A F	Fw	GGAGCAGCGGgctGACTACTACC
FICD S397A R	Rv	TTGCGGATGGTGATGGGC

FICD E394A F	Fw	CATCCGCAAGgctCAGCGGTCCGAC
FICD E394A R	Rv	GTGATGGGCGGGTAGCCC
FICD R396A F	Fw	CAAGGAGCAGgctTCCGACTACTACCACGTGTTGG
FICD R396A R	Rv	CGGATGGTGATGGGCGGG
FICD 187-458 to pAc F	Fw	atgctgctgctgctgagcgcggcgcgcgcggtggaagagatcgaccagaggtatttcag
FICD 187-458 to pAc R	Rv	GCCaccCTTCTCAAACTGCGGATGAGACCAtccgctgggcttcacagg
FICD 102-458 to pAc F	Fw	atgctgctgctgctgagcgcggcgcgcgcgggtaagttggaagccagagctgc
FICD 102-458 to pAc R	Rv	GCCaccCTTCTCAAACTGCGGATGAGACCAtccgctgggcttcacagg
Linearize pAc F	Fw	AAAGATGAGTTGTGAGCGGCCGCGAC
Linearize pAc R	Rv	cgcgcgcgccgcgct
KDEL to GFP F	Fw	CTTGTACAGCTCATCCATGCC
KDEL to GFP R	Rv	AAAGATGAGTTGTGAGCGGCCGCGAC

Supplementary Table 3

Supplementary Table 3. Primers for BiP constructs and mutagenesis

Primers for BiP constructs and mutagenesis	Spalte1	Sequence 5' - 3'
BiP T518A F	Fw	AGACAAAGGTgCAGGGAACAAAAAC
BiP T518A R	Rv	TCAGCTGTCACTCGAAGAATA
BiP T229A F	Fw	GGGCGGTGGAgCCTTCGATGT
BiP T229A R	Rv	AGGTCAAAAACGAGGATGTTCTTCTC
BiP V415A F	Fw	AGGTGATCTGgccCTGCTTGATG
BiP T415A R	Rv	GTATCTTGATCACCAGAG
BiP L417A F	Fw	TCTGGTACTGgccGATGTATGTCCTC
BiP L417A R	Rv	TCACCTGTATCTTGATCAC
BiP R197A F	Fw	GAATGTCATGgccATCATCAATGAGCCCACAG
BiP R197A R	Rv	AGTCCAGCAATGGTGCCA
BiP E217A F	Fw	GAGAGAGGGCgccAAGAACATCC
BiP E217A R	Rv	TTATCCAGGCCATACGCA
BiP D238A F	Fw	TCTGACCATTgccAATGGTGTCTTTG
BiP D238A R	Rv	AGAGACACATCGAAGGTTC
BiP V241A F	Fw	TGACAATGGTgccTTTGAAGTGG
BiP V241A R	Rv	ATGGTCAGAAGAGACACATC
BiP R492A F	Fw	TCCTGCTCCTgcgGGGGTACCCC
BiP R492A R	Rv	GGAATTCCAGTCAGATCAAATG
BiP E514A F	Fw	AGTGACAGCTgcgGACAAAGGTAC
BiP E514A R	Rv	CGAAGAATACCATTAACATC
BiP K516A F	Fw	AGCTGAAGACgcgGGTACAGGGAAC
BiP K516A R	Rv	GTCACTCGAAGAATACCATTAAC
BiP K521A F	Fw	TACAGGGAACgcgAACAAAATCACAATTAC
BiP K521A R	Rv	CCTTTGTCTTCAGCTGTC
BiP 19 -549 F	Fw	TAGTAACTCGAGGCATGCG
BiP 19 -549 R	Rv	AGCAAACTTCTCtGCATCA
BiP 28 -549 F	Fw	ACGGTGGTCGGCATCGAC
BiP 28 -549 R	Rv	GCCCTGAAAATACAGGTTTTCGG

Supplementary References

[1] Drozdetskiy, A., Cole, C., Procter, J. & Barton, G. J. JPred4: A protein secondary structure prediction server. Nucleic Acids Res. **43**, W389–W394 (2015).
