# Supplementary material for: Specificity of AMPylation of the human chaperone BiP is mediated by TPR motifs of FICD
Source: Nat Commun. 2021 Apr 23;12:2426. doi: 10.1038/s41467-021-22596-0 (PMC8065156; doi:10.1038/s41467-021-22596-0)
Supplement: Supplementary file 3 — Reporting Summary [file 41467_2021_22596_MOESM3_ESM.pdf]

nature research

Corresponding author(s): Aymelt Itzen
Christian Hedberg

Last updated by author(s): Mar 11, 2021

Reporting Summary

Nature Research wishes to improve the reproducibility of the work that we publish. This form provides structure for consistency and transparency in reporting. For further information on Nature Research policies, see our Editorial Policies and the Editorial Policy Checklist.

Statistics

For all statistical analyses, confirm that the following items are present in the figure legend, table legend, main text, or Methods section.

n/a	Confirmed	
☐	☒	The exact sample size (*n*) for each experimental group/condition, given as a discrete number and unit of measurement
☐	☒	A statement on whether measurements were taken from distinct samples or whether the same sample was measured repeatedly
☐	☒	The statistical test(s) used AND whether they are one- or two-sided<br>*Only common tests should be described solely by name; describe more complex techniques in the Methods section.*
☒	☐	A description of all covariates tested
☒	☐	A description of any assumptions or corrections, such as tests of normality and adjustment for multiple comparisons
☐	☒	A full description of the statistical parameters including central tendency (e.g. means) or other basic estimates (e.g. regression coefficient) AND variation (e.g. standard deviation) or associated estimates of uncertainty (e.g. confidence intervals)
☐	☒	For null hypothesis testing, the test statistic (e.g. *F*, *t*, *r*) with confidence intervals, effect sizes, degrees of freedom and *P* value noted<br>*Give P values as exact values whenever suitable.*
☒	☐	For Bayesian analysis, information on the choice of priors and Markov chain Monte Carlo settings
☒	☐	For hierarchical and complex designs, identification of the appropriate level for tests and full reporting of outcomes
☒	☐	Estimates of effect sizes (e.g. Cohen's *d*, Pearson's *r*), indicating how they were calculated

Our web collection on statistics for biologists contains articles on many of the points above.

Software and code

Policy information about availability of computer code

Data collection	Gel images and western blots were recorded with ChemoStar Touch v0.5.65 (Intas Science Imaging). HPLC data was recorded with Lab solutions v.5.51 (Shimadzu). Intact mass spectrometry data were collected with either Agilent MassHunter Data Aquisition vB.06.01 (Agilent) or Bruker Compass HyStar 5.0 SR1 v5.0.37.0. CD spectra were collected with Chirascan Spectrometer Control Panel Application Version v4.7.0.194. The melting curves were collected with PR.ThermControl v2.1.2.
Data analysis	Crystallography data was analyzed with DIALS v1.14.5, CCP4 v7.0.078 (incl. Buccaneer, Refmac), PHASER v2.8.3, COOT v0.8.9.2, PHENIX v1.16, MoRDa v1.2.17. Atomic distances were calculated with PyMol v2.3.2 (Schrödinger). Two-tailed paired t test was done with Prism v8.3.1 (GraphPad). Bands in western blots and gels were quantified with Image Lab v6.0.1. Kinetics data fits were made with Origin Pro v9.1 (OriginLab). HPLC data was analyzed with Lab Solutions v5.51 (Shimadzu). Intact mass spectrometry deconvolution was performed with Agilent MassHunter Qualitative Analysis vB.07.00 (Agilent) or Bruker Compass DataAnalysis v5.1. MS/MS data was analyzed by Proteome Discoverer v2.0 (Thermo Scientific). MD simulations were performed using the AMBER software package (v18). The AMBER force field parameters can be found in the AMBER parameter database (http://amber.manchester.ac.uk). The trajectories in MD simulation were processed and analyzed with CPPTRAJ v4.14.0. CD spectra were analyzed via Pro-Data Viewer v4.2.13.

For manuscripts utilizing custom algorithms or software that are central to the research but not yet described in published literature, software must be made available to editors and reviewers. We strongly encourage code deposition in a community repository (e.g. GitHub). See the Nature Research guidelines for submitting code & software for further information.

Data

Policy information about availability of data

All manuscripts must include a data availability statement. This statement should provide the following information, where applicable:

- Accession codes, unique identifiers, or web links for publicly available datasets
- A list of figures that have associated raw data
- A description of any restrictions on data availability

Atomic coordinates and structure factors of the FICDTReND-BiP complex have been deposited in the Protein Data Bank with accession code 6ZMD [https://www.rcsb.org/structure/unreleased/6ZMD]. The mass spectrometry proteomics data have been deposited to the ProteomeXchange Consortium [http://proteomecentral.proteomexchange.org/] via the PRIDE partner repository with the dataset identifier PXD022869 and 10.6019/PXD022869 [ProteomeXchange Dataset PXD022869]. The AMBER force field parameters can be found in the AMBER parameter database (http://amber.manchester.ac.uk). The data that support the findings of this study are available from the corresponding author upon reasonable request. Other Source data are provided with this paper.

Field-specific reporting

Please select the one below that is the best fit for your research. If you are not sure, read the appropriate sections before making your selection.

- [x] Life sciences
- [ ] Behavioural & social sciences
- [ ] Ecological, evolutionary & environmental sciences

For a reference copy of the document with all sections, see nature.com/documents/nr-reporting-summary-flat.pdf

Life sciences study design

All studies must disclose on these points even when the disclosure is negative.

Sample size	None of the statistical method was used to predetermine sample size. To ensure reproducibility of the findings, for all in vitro assays, at least three independent biological replicates were performed (n=3). Further details of sample size are included in corresponding figure legend and methods.
Data exclusions	No data were excluded.
Replication	All data presented in the manuscript are reproducible. Numbers of replications for each experiment are stated in the corresponding figure legends.
Randomization	Not relevant for the experimental design.
Blinding	Not relevant for the experimental design.

Reporting for specific materials, systems and methods

We require information from authors about some types of materials, experimental systems and methods used in many studies. Here, indicate whether each material, system or method listed is relevant to your study. If you are not sure if a list item applies to your research, read the appropriate section before selecting a response.

Materials & experimental systems

n/a	Involved in the study	
☐	☒	Antibodies
☐	☒	Eukaryotic cell lines
☒	☐	Palaeontology and archaeology
☒	☐	Animals and other organisms
☒	☐	Human research participants
☒	☐	Clinical data
☒	☐	Dual use research of concern

Methods

n/a	Involved in the study	
☒	☐	ChIP-seq
☒	☐	Flow cytometry
☒	☐	MRI-based neuroimaging

Antibodies

Antibodies used	Monoclonal mouse α-AMP antibodies (1:1000 dilution, Hoepfner et al. 2020; doi: 10.1016/j.isci.2020.101800),<br>Rabbit α-GFP primary antibody (A11122; Life Technologies, 1:2000 dilution),<br>Rabbit α-GRP78 primary antibody (PA5-34941; Thermo Scientific; 1:5000 dilution),<br>Mouse α-GAPDH primary antibody (sc-47724; Santa Cruz; 1:1000)<br>Goat secondary α-rabbit-HRP-antibody (12-348, Sigma, 1:40000 dilution)<br>Goat secondary α-mouse-HRP-antibody (31430, Thermo Scientific, 1:20000 dilution)

Validation	Validation for α-AMP antibody has been published previously (Hoepfner et al. 2020; doi: 10.1101/2020.06.23.164731, validated for sensitive and specific recognition of AMPylation independent of protein backbone in western blots of both mammalian cell lysates and purified proteins),<br><br>Rabbit α-GFP primary antibody (validated for WB, IF, ICC, IHC, IP; tag specific; Life Technologies). Validated via specific recognition of GFP in lysates of GFP-transfected HeLa cells in western blots.<br><br>Rabbit α-GRP78 primary antibody (validated for WB, IF, ICC, IHC; GRP78 specific; Thermo Scientific). Validated via specific recognition of endogenous BiP in lysates of various cell types via western blot.<br><br>Mouse α-GAPDH primary antibody (validated for WB, IF, IP, IHC; GAPDH specific; Santa Cruz). Validated via specific recognition of endogenous GAPDH in lysates of various human cell types via western blot.<br><br>Goat secondary α-rabbit-HRP-antibody (validated for WB, ELISA, IHC; rabbit specific, Sigma). Validated by the supplier for usage in WB, ELISA and IHC.<br><br>Goat secondary α-mouse-HRP-antibody (validated for WB, ELISA, ICC, IHC, IP; mouse specific; Thermo Scientific). Specific recognition of primary mouse antibody is validated by the supplier by detection of diverse model proteins (e.g. tubulin, CD38...) in diverse cell lysates with a specific mouse antibody and goat secondary α-mouse-HRP-antibody.

Eukaryotic cell lines

Policy information about cell lines

Cell line source(s)	All cell lines used in this study are commonly used cell lines that were available by in-house stocks. HEK293 cell line (DSMZ ACC-305)
Authentication	Authentication is performed by providers. No additional authentication has been peformed in our labs.
Mycoplasma contamination	Cells were tested for mycoplasma contamination and cells were mycoplasma negative.
Commonly misidentified lines (See ICLAC register)	No commonly misidentified cell lines were used.
